# Editorial

**DOI:** 10.1007/s41682-021-00098-6

**Published:** 2021-12-13

**Authors:** Olaf Müller

**Affiliations:** Institut für Soziologie, Universität Münster, Münster, Deutschland

Mit dieser Ausgabe vollendet die ZRGP das fünfte Jahr ihres Erscheinens und feiert damit immerhin schon einmal das kleinstmögliche Jubiläum. Und man darf wohl sagen: Die Zeitschrift hat sich etabliert und wurde gut angenommen – sowohl durch Autorinnen und Autoren, als auch, was die Leserschaft betrifft. Das inhaltliche Profil mit dem sozialwissenschaftlich orientierten Fokus auf die Verschränkung und das Zusammenspiel von Religion bzw. Religiosität mit gesellschaftlichen und politischen Fragestellungen, Konflikten und Konstellationen ist, so denken wir, deutlich zu erkennen. In formal-struktureller Hinsicht lässt sich eine gewisse Veränderung zu den ersten Heften dahingehend feststellen, dass die ZRGP einem Trend folgt, der bei vielen anderen Fachzeitschriften ebenfalls zu beobachten ist, nämlich von Zeit zu Zeit ein ganzes Heft einem bestimmten Thema zu widmen oder auch innerhalb eines Heftes *Special Sections* zu gestalten. Die Vorteile der Vorgehensweise, thematisch verwandte Beiträge örtlich und zeitlich gebündelt zu präsentieren, liegen auf der Hand – zumal hierbei selbstverständlich die gleichen Qualitätsstandards greifen, die wir für jeden einzelnen Beitrag auch zugrunde legen. Wir als Redaktion würden uns jedenfalls freuen, wenn uns auch weiterhin entsprechend systematisch angelegte Exposés dieser Form erreichen.

Ein Blick auf die bisherigen Hefte wie auch in die aktuelle Ausgabe genügt jedoch, um sich vor Augen zu führen, wie sehr die Zeitschrift auch von den Beiträgen lebt, die nicht in einen übergreifenden thematischen Rahmen eingebunden sind. Diese Einzelartikel laufen ganz sicher auch in Zukunft nicht Gefahr zum Füllmaterial degradiert zu werden, sondern werden nach wie vor ihren festen und gleichberechtigten Platz haben. In diesem Sinne möchten wir an dieser Stelle ebenfalls dazu ermuntern, uns auch weiterhin thematisch einschlägige Manuskripte dieser Art zukommen zu lassen.

Die drei jüngsten Ausgaben stehen exemplarisch für das Bemühen, die verschiedenen Formate alle zu ihrem Recht kommen zu lassen: Nach einer offenen Nummer (November 2020) und einem *Special Issue* zum Thema „Religion und Populärkultur“ (jüngst im November 2021) erscheint nun ein gemischtes Heft mit einer *Special Section *zum Thema „Religion und Rechtsextremismus“ sowie vier Einzelbeiträgen. Da für erstere seitens der beiden Herausgeber *Kornelia Sammet* und *Alexander Yendell* ein eigenes Editorial verfasst wurde, muss an dieser Stelle nicht näher darauf eingegangen werden. Stattdessen seien hier die Einzelbeiträge kurz vorgestellt, die sich nicht nur recht unterschiedlichen Thematiken widmen, sondern auch methodisch und konzeptuell ganz verschiedene Zugänge wählen. Wenn man einen gemeinsamen Nenner suchen möchte, dann wäre er wohl darin zu sehen, dass in allen Beiträgen (wie auch bei den Abhandlungen in der *Special Section*) das Konfliktpotential von Religion bzw. Religiosität thematisiert wird.

*Annette Schnabel* und *Lisa Hönes* wenden sich in ihrem Beitrag auf der Basis von explorativen quantitativen Dokumentenanalysen von Verfassungstexten aus 186 Ländern der Frage zu, inwieweit und in welchem Ausmaß Identitätsangebote, die Verfassungen den Bürgerinnen und Bürgern unterbreiten, auf Religion bzw. Religiosität Bezug nehmen. Ist das der Fall, kann das zwar zunächst einmal als Versuch aufgefasst werden, Religion als gemeinschaftsbildendes Element zu definieren. Durch die Tatsache, dass damit fast notwendigerweise auch bestimmte Exklusionstendenzen verbunden sind, ist das Konfliktpotential aber hier ebenfalls inhärent. Die Autorinnen analysieren zur Beantwortung ihrer Fragestellung entsprechende Formulierungen in den Präambeln der Verfassungen, den Bestimmungen hinsichtlich der Gewährung und Einschränkung individueller Freiheiten sowie bestimmter Regelungen zur religiösen Unterweisung und kommen dabei zu dem Schluss, dass der Religion in allen untersuchten Themenfeldern weltweit eine wichtige Rolle zukommt.

Aus ebenfalls quantitativer Perspektive, allerdings anhand von Individualdaten, untersucht *Cemal Öztürk*, in welchem Ausmaß, von wem und warum muslimischen Bürgermeisterkandidaten in Deutschland Skepsis und Ablehnung entgegengebracht werden. Was das Konfliktpotential der Religion in dieser Frage betrifft, so zeigt sich dieses schon in der Tatsache, dass allein der spezifische religiöse Hintergrund der fiktiven Bürgermeisterkandidaten offenbar ausreicht, um bei der Mehrheit der Befragten Vorbehalte auszulösen. Aber auch auf Seiten der Befragten stellt sich ein bestimmter religiöser Background (Mitgliedschaft in evangelisch-freikirchlichen bzw. orthodoxen Gemeinschaften) als signifikanter Faktor heraus, der Skepsis und Ablehnung befördert.

Im konzeptuell ausgerichteten Aufsatz von *Katharina Heyden* und *Martino Mona* taucht der Konflikt bereits im Titel auf. Ausgehend von der Überlegung, dass Konflikte, die eine religiöse Aufladung erfahren, durch die damit verbundene Emotionalisierung erheblich schwerer zu lösen sind bzw. sich einer Lösung ganz entziehen, zeigt der Beitrag einen Weg auf, der dieses kaum zu erreichende Ziel gar nicht erst anstrebt, sondern auf Transformation und Bewältigung setzt. Der im Rahmen einer interdisziplinären Forschungskooperation entwickelte Ansatz basiert auf dem psychologischen Prinzip des *Coping*, welches im Sinne eines *travelling concept* von den Autoren als besonders fruchtbar für die Analyse von religiösen Konflikten angesehen wird.

Der offene Teil des Heftes schließt mit dem ersten Beitrag in dieser Zeitschrift zum Thema Covid-19. Dass hier schon aufgrund des Themengegenstandes der Konflikt mitschwingt, bedarf wohl keiner näheren Erläuterung. *Robert Schäfer* und *Nadine Frei* stellen in ihrem Beitrag die Ergebnisse von qualitativen Interviews vor, die sie mit Personen geführt haben, die die staatlichen Corona-Maßnahmen kritisieren. Sie zeigen dabei auf, dass diese Kritik einem rationalistischen Verständnis von Krisenlösung entspringt, welches auf der Annahme beruht, es müsse „perfekt“ rationale Lösungen für eine solche Krise geben. Dem von den Autoren beobachteten Grundmuster der Kritik, der Kombination aus verschwörungstheoretischen und esoterischen Deutungsmustern (*conspirituality*), wohnt jedoch selbst ein irrationales Element inne.

